# Eco-friendly approach to access of quinoxaline derivatives using nanostructured pyrophosphate Na_2_PdP_2_O_7_ as a new, efficient and reusable heterogeneous catalyst

**DOI:** 10.1186/s13065-020-0662-z

**Published:** 2020-02-03

**Authors:** Karim Dânoun, Younes Essamlali, Othmane Amadine, Hassan Mahi, Mohamed Zahouily

**Affiliations:** 1 Moroccan Foundation for Advanced Science, Innovation and Research (MAScIR), VARENA Center, Rue Mohamed El Jazouli, Madinat Al Irfane, 10100 Rabat, Morocco; 2 University Hassan II Casablanca, FST Mohammedia, Laboratory of Materials, Catalysis and Valorization of Natural Resources - URAC 24, B.P. 146, 20650 Casablanca, Morocco

**Keywords:** Nanostructured pyrophosphate, Heterogeneous catalysis, Recyclable catalyst, Quinoxalines, 1,2-Diamine, 1,2-Dicarbonyl

## Abstract

In the present study, we report the synthesis of various quinoxaline derivatives from direct condensation of substituted aromatic 1,2-diamine with 1,2-dicarbonyl catalyzed by nanostructured pyrophosphate Na_2_PdP_2_O_7_ as a new highly efficient bifunctionalheterogeneous catalyst. The quinoxaline synthesis was performed in ethanol as a green and suitable solvent at ambient temperature to afford the desired quinoxalines with good to excellent yields in shorter reaction times. Many Quinoxaline derivatives were successfully synthesized using various 1,2-diketones and 1,2-diamines at room temperature. Catalyst reusability showed that the Na_2_PdP_2_O_7_ catalyst exhibited excellent recyclability without significant loss in its catalytic activity after five consecutive cycles. 
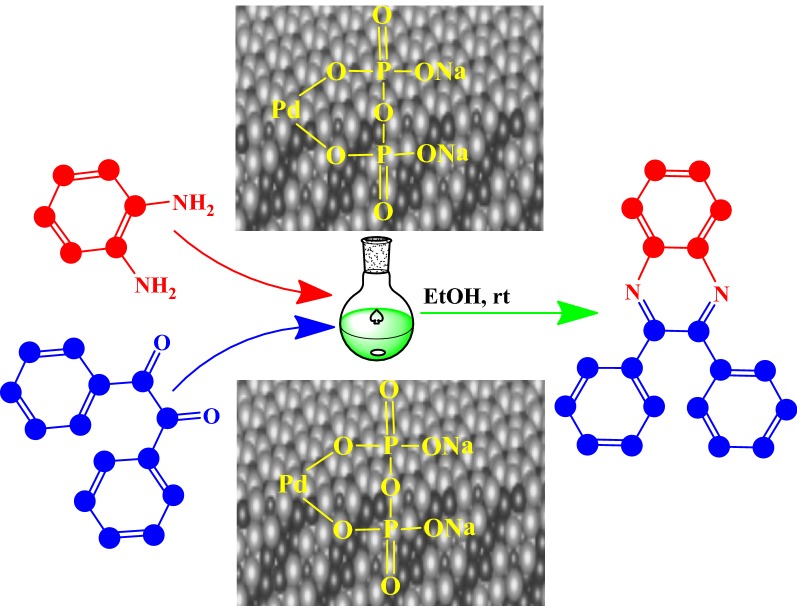

## Introduction

Quinoxaline and its derivatives are an important class of heterocyclic compounds, they have attracted considerable attention over the years owing to their very interesting pharmaceutical and biological properties such as insecticidal, antifungal, anthelmintic, anticancer, antibacterial and antiviral [1–6]. Beside their medicinal applications, these compounds have been widely used as dyes, electroluminescent materials, photo-initiators and also in organic semiconductors [7–10]. Recently, much more attention has been devoted to the development of sustainable and efficient methods for the synthesis of quinoxalines derivatives. Over the years, several synthetic strategies have been reported in literature for the preparation of substituted quinoxalines compounds, some example include the oxidative coupling of epoxides and ene-1,2-diamines [11], the reductive cyclization of 1,2-dicarbonyl compounds with 2-nitroanilines [12], the oxidative cyclization of α-hydroxyketones with *o*-phenylenediamines [13], the coupling of α-diazoketones with aryl 1,2-diamines [14], the reaction of α-haloketones with aromatic 1,2-diamines [15], the intramolecular cyclization of dialdimines [16], and the reaction of aryl-1,2-diamines and diethyl bromomalonate [17]. Furthermore, quinoxaline and its derivatives can also be successfully synthesized from the direct condensation of aryl 1,2-diamines with 1,2-dicarbonyl compounds. Currently, the synthesis of quinoxaline derivatives is usually carried out in the presence of a variety of catalysts. The most commonly used catalysts are polyaniline sulfate salt [18], oxalic acid [19], cerium(IV) ammonium nitrate [20], sulfamic acid [21], Wells–Dawson heteropolyacid [22], bismuth(III) triflate [23], indium chloride [24], ionic liquid 1-*n*-butylimidazolium tetrafluoroborate [25], zirconium tetrabis(dodecylsulfate) [26], palladium(II)acetate [27], gallium(III) triflate [28] and molecular iodine [29]. However, these catalytic systems suffer from several drawbacks, mainly, the drastic reaction conditions such as, high reaction temperature, high catalyst amount, prolonged reaction time even under microwave or ultrasound irradiation, contamination of the product even after purification, and it is impossible to regain the costly catalyst for reuse [30, 31], as well as the environmental pollution caused by the use of a considerable amount of toxic solvents, thus making the process more complicated, expensive, and environmentally unfriendly. Hence, the development of sustainable protocols to design new reusable and efficient heterogeneous catalytic systems that could be used in cleaner process has attracted tremendous interest, and numerous heterogeneous catalytic systems have been reported to be successful for the synthesis of quinoxaline derivatives. ZnO-KIT-6 [32], Ni-nanoparticles [33], Yb/NaY zeolite [34], Al_2_O_3_ [35], graphene oxide [36], nanocrystalline CuO [37], Nano-TiO_2_ [38], montmorillonite K-10 [18]. Another type of materials based on metal phosphates and pyrophosphates are also good candidates for the catalysis of numerous reactions requiring acidic catalysts. These metal pyrophosphates (MP_2_O_7_) are of a very high interest thanks to their wide range of utilization ranging from ceramics [39] to optical materials [40] and packing materials for chromatographic columns [41]. Among these materials, palladium pyrophosphate has only rarely been explored, for the best of our knowledge, there has not been any report in the literature for the use of a palladium pyrophosphate as a nanocatalyst for the condensation reaction of 1,2-diamine with 1,2-dicarbonyl. Therefore, in continuation of our studies on the development of new efficient synthetic strategies [42, 43], the main objective of the present study is to develop a green and simple route for the synthesis of quinoxaline derivatives from direct condensation between 1,2-diamines and 1,2-dicarbronyl compounds in green solvent at room temperature eover the nano structured Na_2_PdP_2_O_7_ as a novel heterogeneous catalyst. Furthermore, the structural, textural, surface and morphological properties of the prepared nanocatalysts, reaction conditions and the nanocatalyst reusability were carefully studied.

## Experimental

### Materials

All the chemicals are purchased commercially and used without any further purification. The Palladium chloride (PdCl_2_), sodium phosphate monobasic dehydrate (NaH_2_PO_4_∙2H_2_O), Absolute alcohol, Dichloromethane, Acetonitrile and Ethyl acetate were purchased from Aldrich chemicalcompany.

### Structural characterization

FTIR spectra of the catalyst were recorded using an ABB Bomem FTLA 2000 spectrometer equipped with a Golden Gate single reflection ATR accessory. Thermal behavior of sample was studied by Thermogravimetric Analysis (TGA) using a Q500 instrument (TA Instruments) with heating rate 10 °C/min, under air atmosphere. X-ray diffraction (XRD) patterns were acquired on a Bruker AXS D-8 diffractometer using Cu K_α_ source (λ = 1.5418 Å), operating in Bragg–Brentano geometry (θ–2θ). The SEM micrographs were obtained using FEI Quanta 200 microscope equipped with EDX detector. Transmission electron micrographs were obtained using a FEI microscope operating at accelerating voltage of 120 kV. The specific surface areas were determined from the nitrogen adsorption/desorption isotherm (at − 196 °C) using the BET (Brunauer–Emmett–Teller) method. The N_2_ adsorption–desorption isotherm data was collected using a Micromeritics 3Flex surface characterization analyzer. Pore size distribution was determined from the N_2_ adsorption isotherm according to the Barret, Joyney and Halenda (BJH) theory. NMR spectra were recorded at 14 T on a BrukerAvance III 600 MHz NMR spectrometer, with working frequencies of 600.13 and 150.902 MHz for proton and carbon respectively, using CDCl_3_as solvent and TMS as the internal standard. The local chemical structure around phosphorus atoms was analyzed by solid-state ^31^P-nuclear magnetic resonance using magic angle spinning conditions (MAS-NMR) spectroscopy.

### Synthesis of the Na_2_PdP_2_O_7_ catalyst

**Scheme 1:**
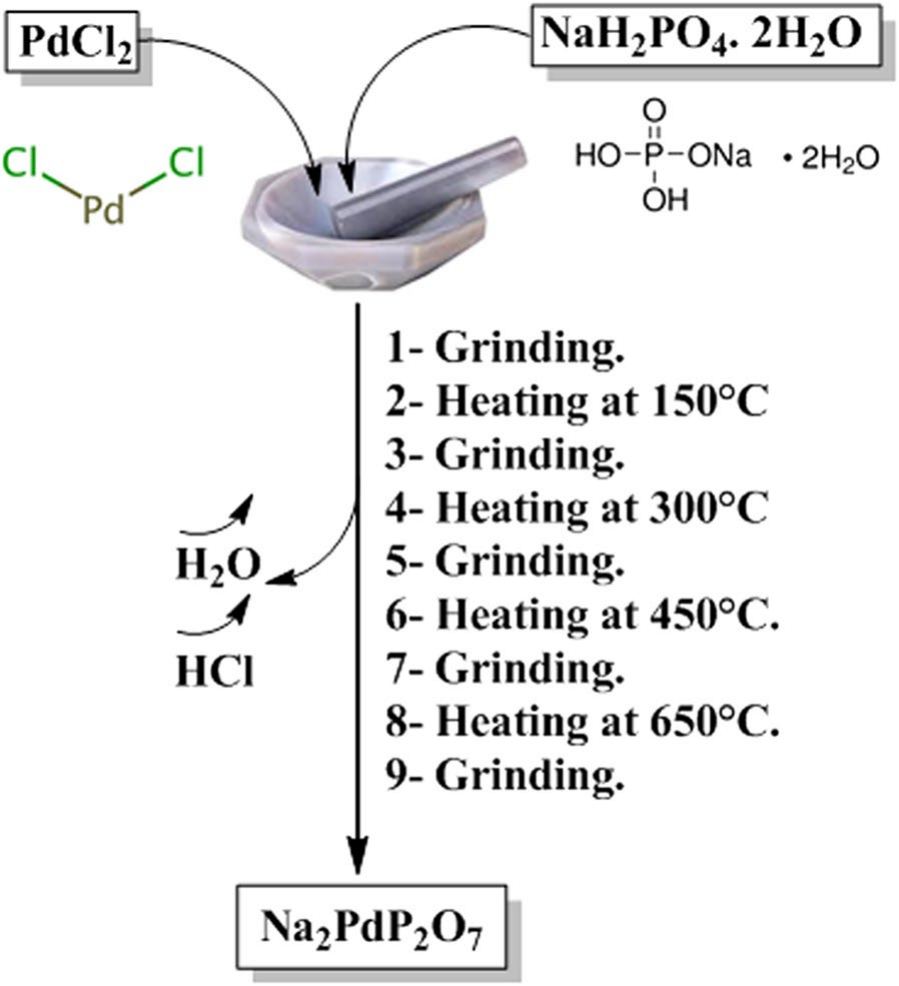
Synthesis of nanostructured pyrophosphate Na_2_PdP_2_O_7_

The nanostructured pyrophosphate Na_2_PdP_2_O_7_ catalyst was prepared by the method recently described in the literature [44], using NaH_2_PO_4_∙2H_2_O and PdCl_2_ as starting materials in a molar ratio of 2:1, respectively. Typically, NaH_2_PO_4_∙2H_2_O and PdCl_2_ were thoroughly mixed by grindingin an agate mortar to insure better contact opportunity between the components. After grinding, the solid powders were progressively heated in an alumina crucible from room temperature to 650 °C at a heating rate of 10 °C/min, and then rapidly quenched according to the procedure described in Scheme 1. Once the thermal treatment was finished, the obtained yellow powder was ground into fine powder.

### General procedure for the preparation of quinoxalines (3a–3h)

Under air atmosphere, an oven-dried round-bottomedflask was charged with equimolar amounts of 1,2-diamine (1 mmol) and 1,2-diketone (1 mmol). Afterward, ethanol (3 mL) and catalyst (10 mg, 3.06 mol.%)were added and the reaction mixturewas stirred at room temperature for 30 min. The reaction progress was monitored by thin layer chromatography (TLC) using Hexane/Ethylacetate (9/1) as eluent. After the completion of the reaction, the catalyst was recovered by simple filtration and then repeatedly washed with dichloromethane. The solvent was evaporated under reduced pressure, and the crude product was purified by simple recrystallization in ethanol to yield the desired product.

## Results and discussion

### Characterization of the catalyst

**Fig. 1:**
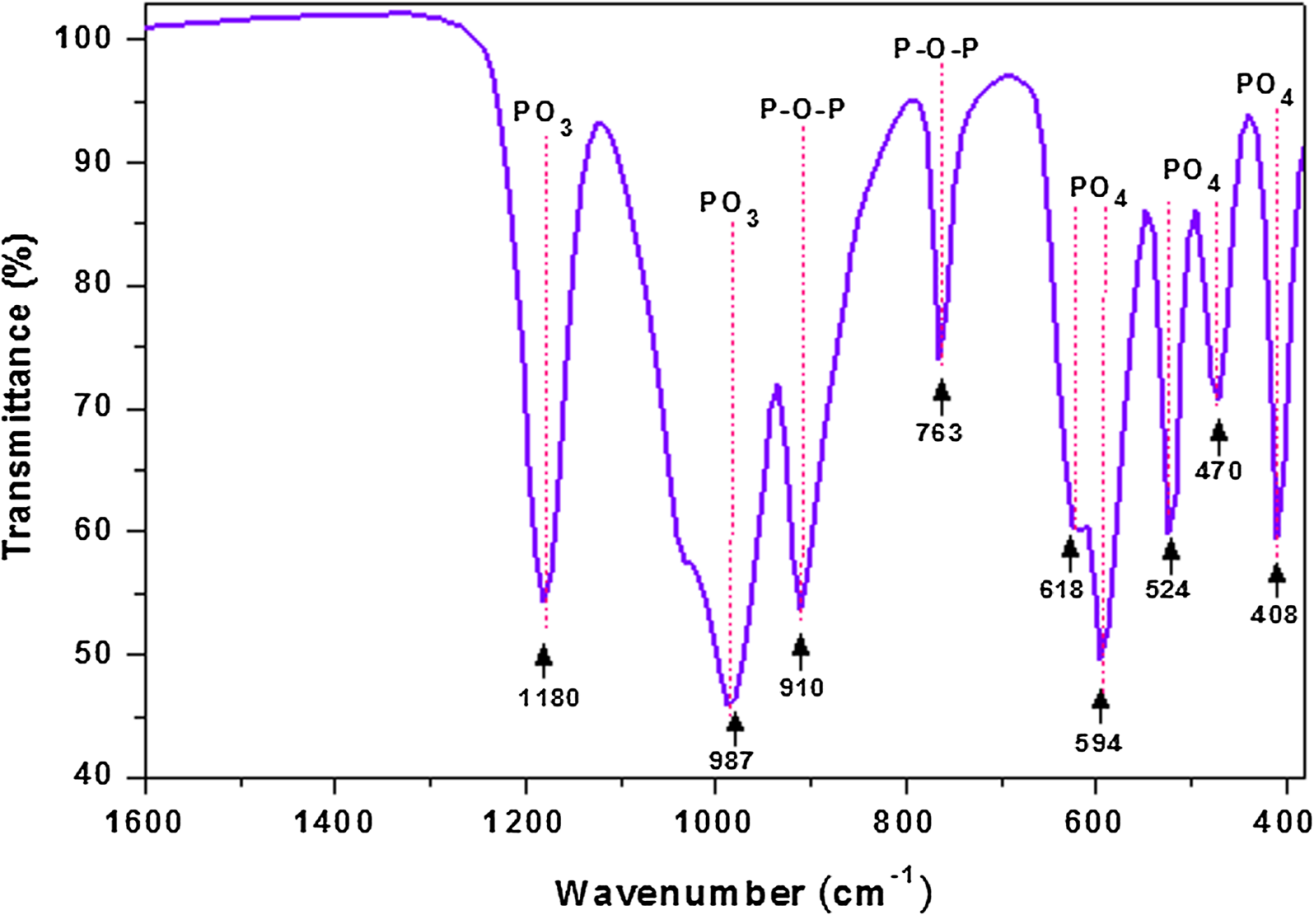
FT-IR spectrum of nanostructured Na_2_PdP_2_O_7_

The FTIR spectrum of the Na_2_PdP_2_O_7_ catalyst is depicted in Fig. 1. As shown in this figure, the bands observed at 1180 and 987 cm^−1^, were assigned to the anti-symmetric and symmetric vibration modes of PO_3_ group, respectively. The strong bonds observed at 763 and 910 cm^−1^ and were attributed to the symmetric and anti-symmetric vibrations bands of P–O–P group. Furthermore, the bands appear at around 400–700 cm^−1^ were assigned to the deformation and rocking modes of PO_4_ group.

**Fig. 2:**
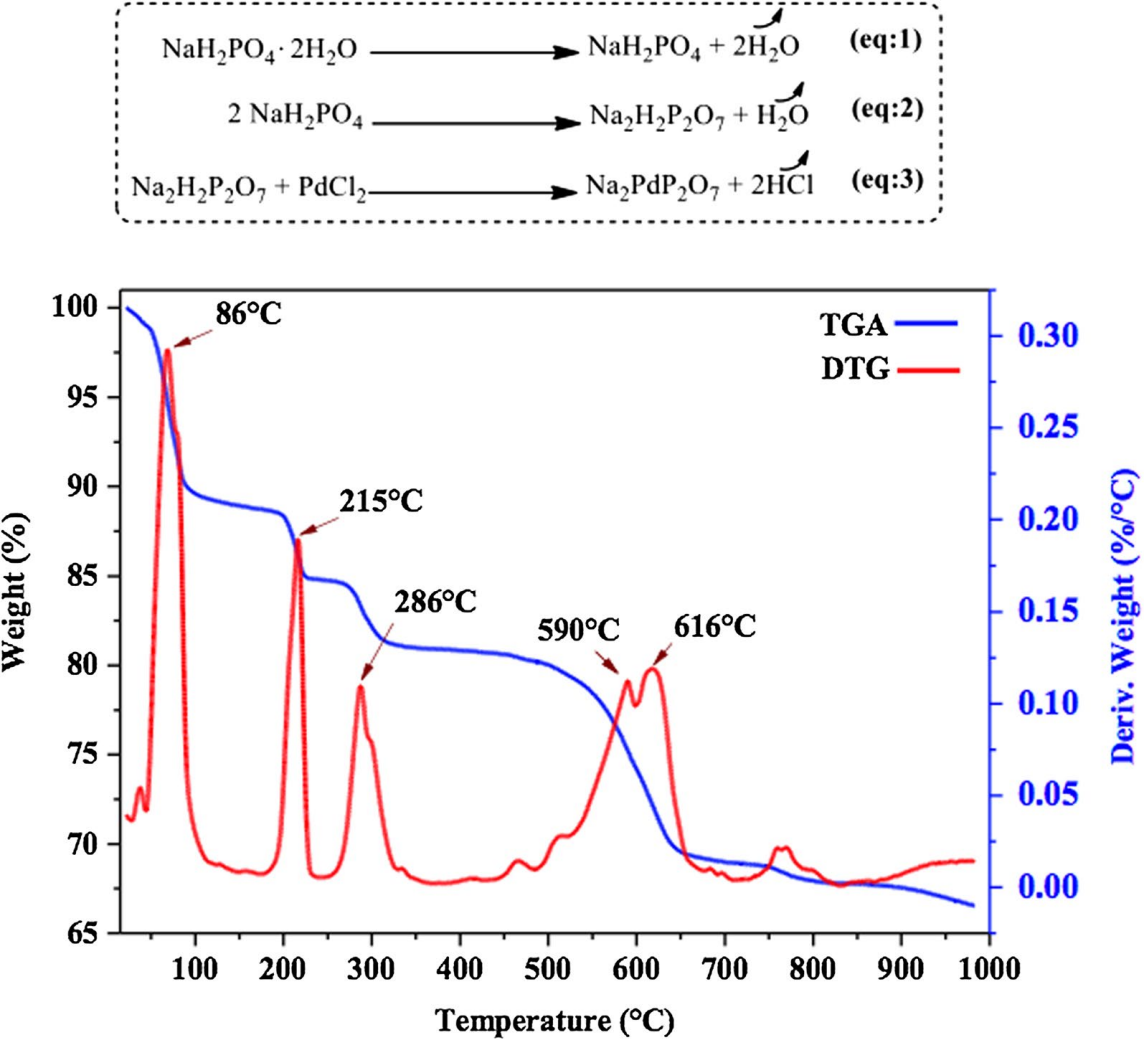
TGA and DTG curves of the mixture of NaH_2_PO_4_∙2H_2_O and PdCl_2_

The TGA/DTG analysis of the solid-state mixture of the starting reagents (NaH_2_PO_4_∙2H_2_O and PdCl_2_) are presented in Fig. 2. According to TGA curve, the mixture of the starting reagents exhibited four consecutive weight losses. The first weight loss observed below 86 °C can be attributed to the removal of the adsorbed water on the surface of the sample. The second weight loss observed at 215 °C, corresponds to the loss of the two molecules of crystal water in NaH_2_PO_4_·2H_2_O (Eq. 1). The third weight loss occurred at the temperature of 286 °C can be assigned to the melting process and the dehydration of NaH_2_PO_4_ as shown in Eq. 2. It is well known that NaH_2_PO_4_ dehydrate to acid pyrophosphate at a temperature higher than its melting points [45]. The last weight loss observed at 590 and 616 °C, which may be related to the reaction of melted alkali metal phosphates with palladium chloride according to Eq. 3.

**Fig. 3:**
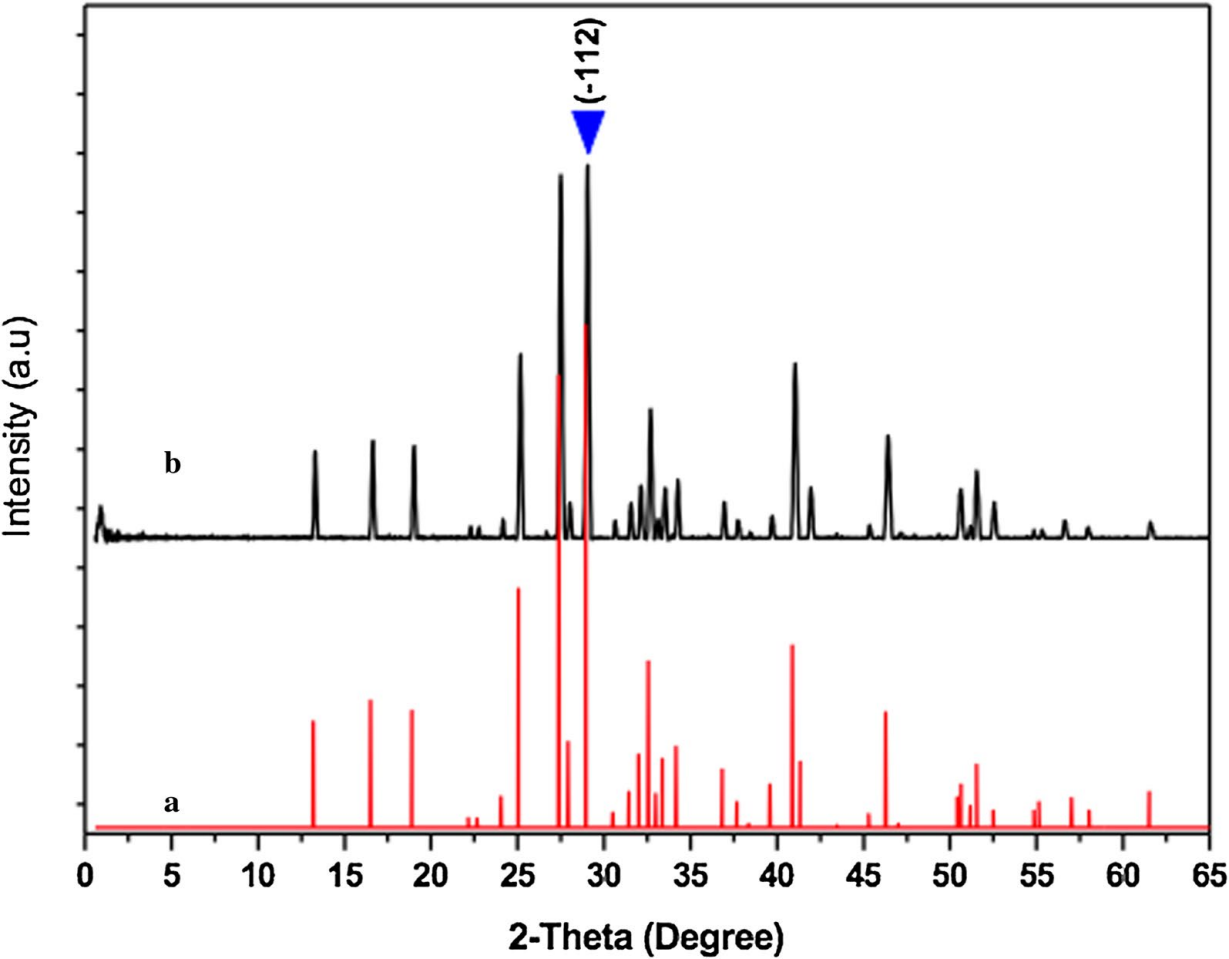
XRD diffraction patterns of Na_2_PdP_2_O_7_ standard (**a**) and product (**b**)

The X-Ray diffraction (XRD) pattern of the as-prepared material is shown in Fig. 3. The XRD pattern of the prepared material indicated that all the diffraction peaks are in good agreement with those of pure Na_2_PdP_2_O_7_ according to the JCPDS file No 10-6543 (Fig. 3a). No typical peaks of impurities were observed in the XRD spectrum, indicating single crystal structure of the as-prepared Na_2_PdP_2_O_7_ catalyst. Moreover, it was observed that the Na_2_PdP_2_O_7_ material exhibited narrow and high peaks suggesting that the as-prepared Na_2_PdP_2_O_7_ is very small in size and has excellent crystallinity. The average crystallite size of the as-prepared Na_2_PdP_2_O_7_ material estimated according to the Scherrer equation is about 7.9 nm.

**Fig. 4:**
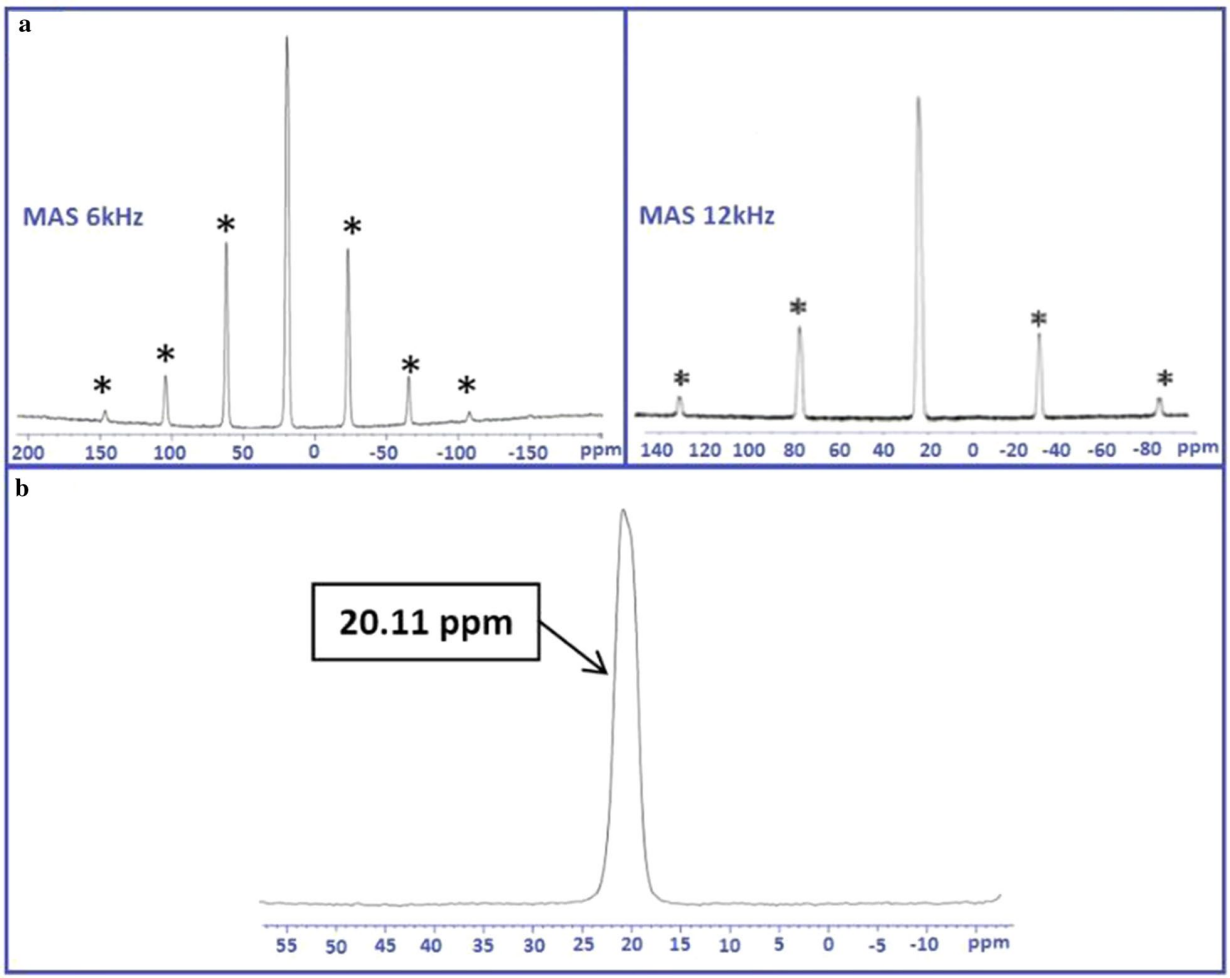
Solid-state ^31^P-MAS NMR spectra of Na_2_PdP_2_O_7_ for frequencies of 6 and 12 kHz **(a)** asterisks indicate the rotation bands. Zoom on the isotropic signal **(b)**

In order to support the aforementioned interpretation ^31^PMAS-NMR studies were also investigated. As shown in Fig. 4 , at a rotation frequency of 6 kHz, the isotropic signal was accompanied with other peaks attributed to the rotation bands on the magic angle spinning spectra of the ^31^P. These bands became more separated when performing measurement at higher rotation frequency (12 kHz). Furthermore, the presence of one single crystallographic site of phosphorus at a chemical shift of δ = 20.11 ppm, proves the existence of only one type of phosphorus site in the Na_2_PdP_2_O_7_ material (Fig. 4b).

**Fig. 5:**
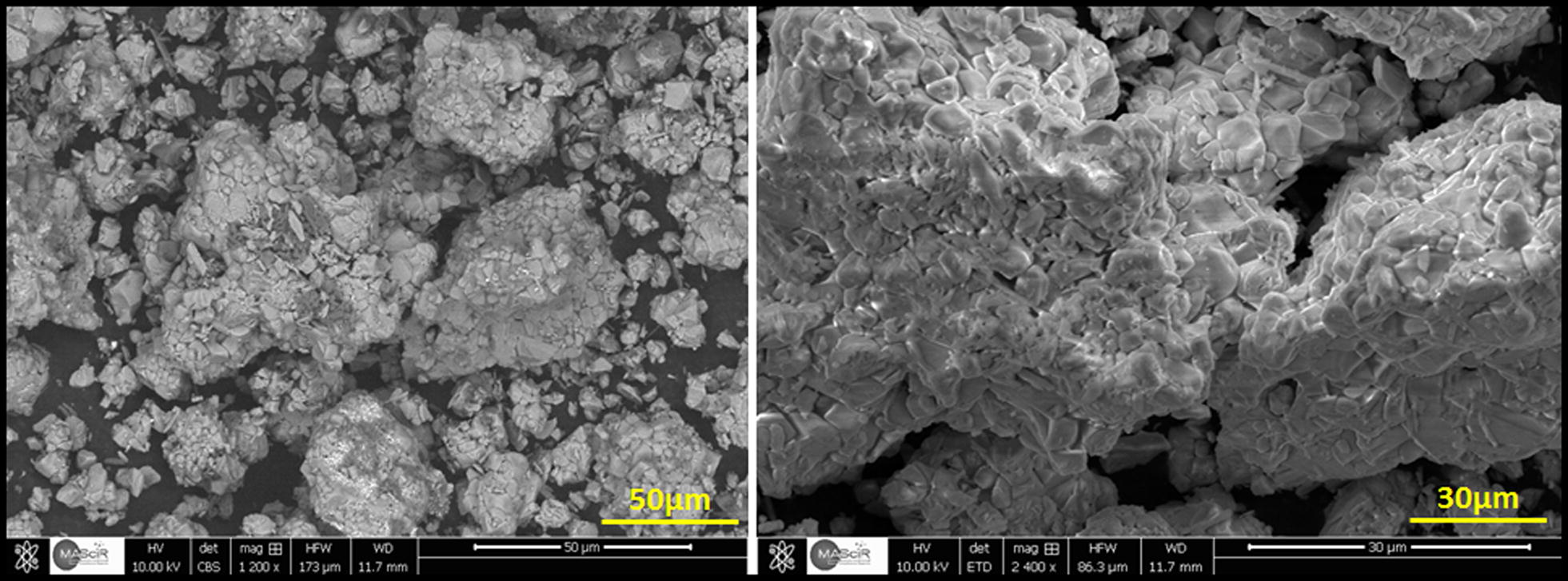
SEM micrographs of nanostructured Na_2_PdP_2_O_7_

The surface morphology of Na_2_PdP_2_O_7_ material was studied by scanning electron microscope (SEM) as shown in Fig. 5. The obtained micrographs showed clearly that the surface of Na_2_PdP_2_O_7_ is homogeneous in size and the shapes and the agglomerates were arranged randomly. Additionally, the surface of these agglomerates is moderately smooth with low visible porosity. This can be explained by a heterogeneous growth of the crystallites caused by the adopted synthesis method, consequently affecting the morphology and porosity.

**Fig. 6:**
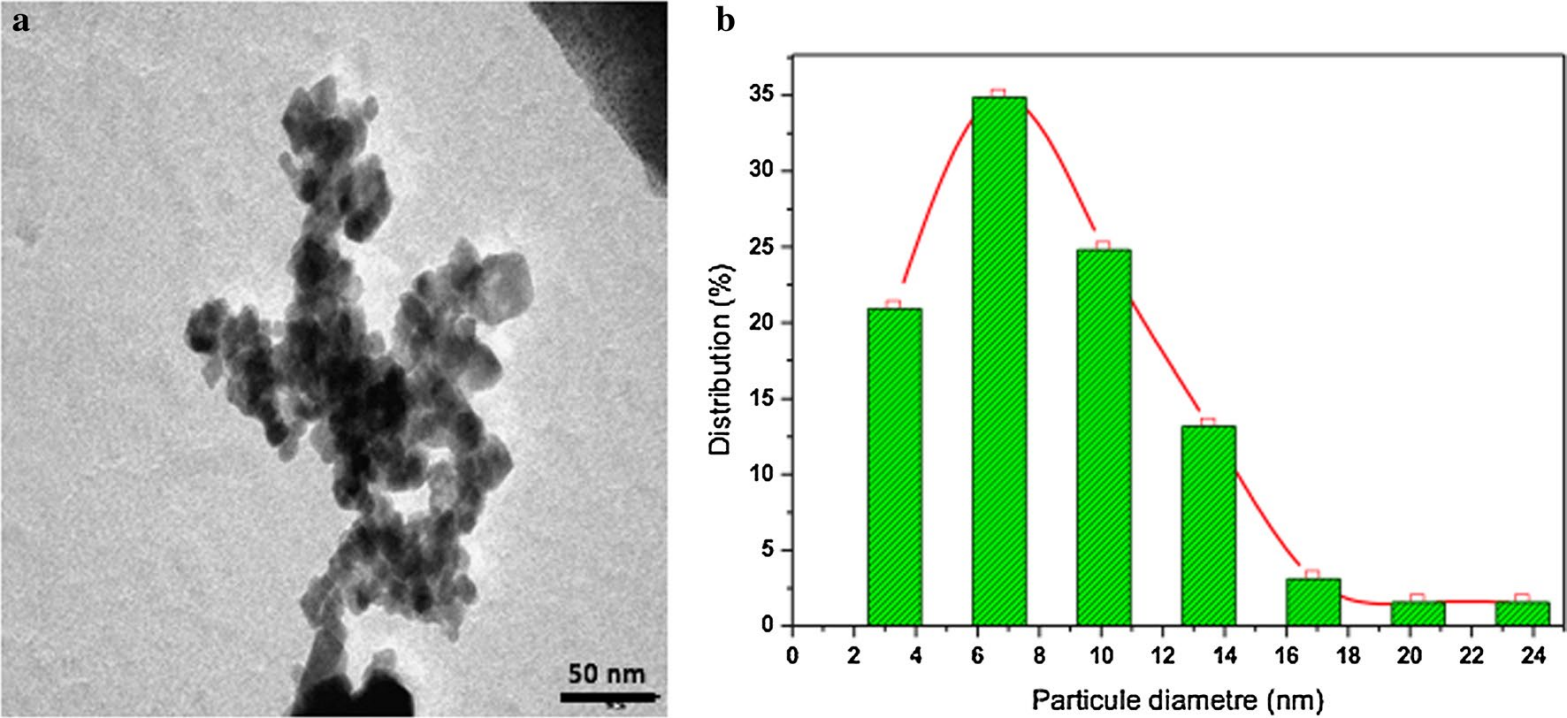
TEM images (**a**), and particle size distribution (**b**) of Na_2_PdP_2_O_7_

The as-prepared Na_2_PdP_2_O_7_ was analyzed by TEM (Fig. 6). The micrograph obtained showed that the Na_2_PdP_2_O_7_ particles were clustered and formed heterogeneous aggregates of nanoparticles that were small in size and irregularly formed (Fig. 6a). By using image J software, the particle size histogram was drawn (from 2.3 to 24 nm), and the mean size of the particles was determined to be around 7 nm (Fig. 6b).

**Fig. 7:**
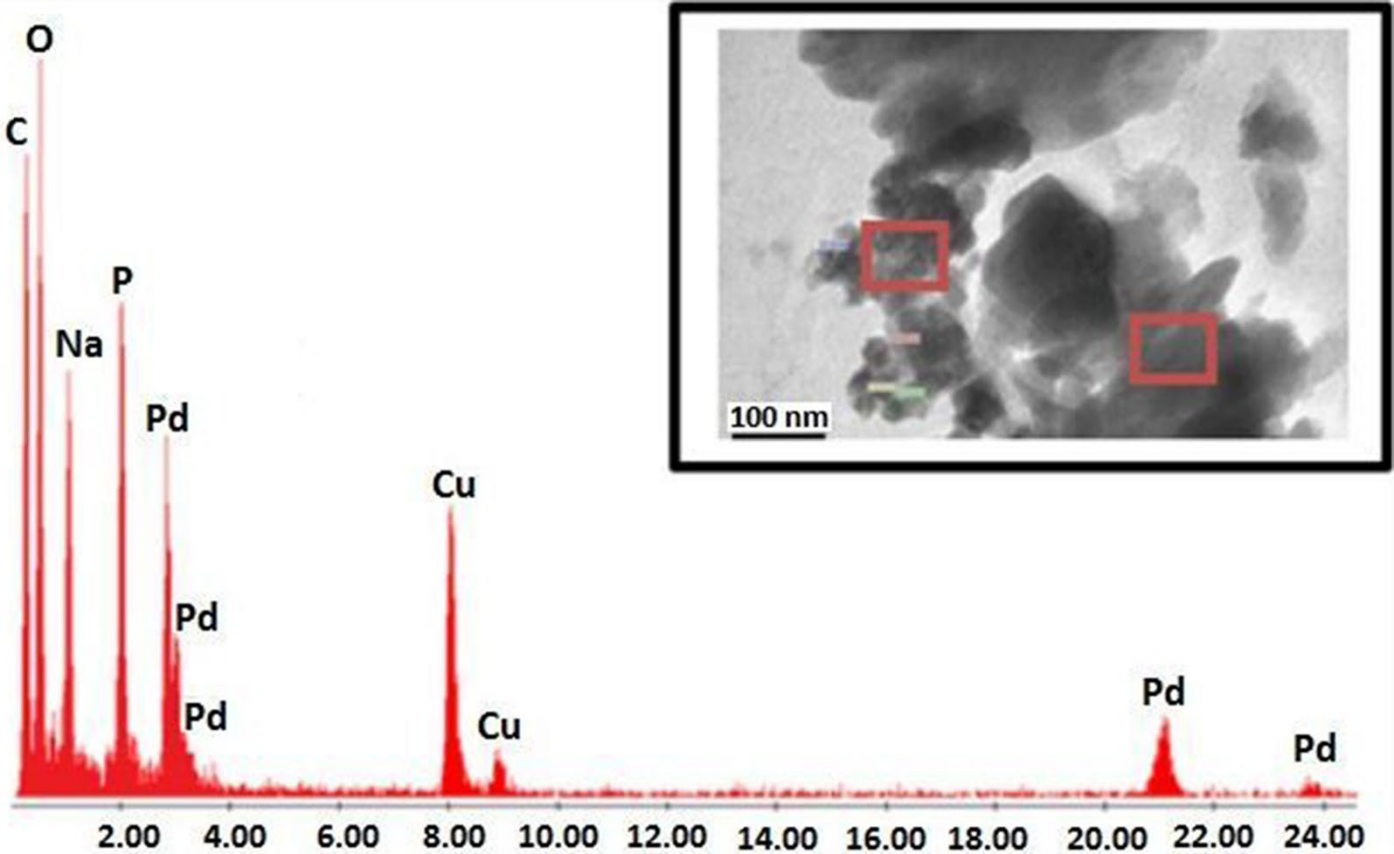
EDS spectrum of Na_2_PdP_2_O_7_ (red square indicates the location of EDS analysis). The result in atomic % is as follow: Na: 17.26; Pd: 7.60; P: 17.54; O: 57.60

The chemical composition of the as-prepared Na_2_PdP_2_O_7_ catalyst was investigated by energy dispersive spectroscopy (EDS). The measurements were performed in two different zones of the sample as shown by the red square in Fig. 7. From EDS analysis, it was confirmed the presence of the characteristic peaks of Na, P, O, and Pd elements in the Na_2_PdP_2_O_7_ material. In addition, the results in relative atomic percentages of these elements were found to be closed to those calculated theoretically. The result in atomic % is as follow: Na: 17.26; Pd: 7.60; P: 17.54; O: 57.60. In addition, no trace of any impurity was detected in EDS spectrum of Na_2_PdP_2_O_7_. It is interesting to note that the C and Cu peaks come from the TEM grid.

**Fig. 8:**
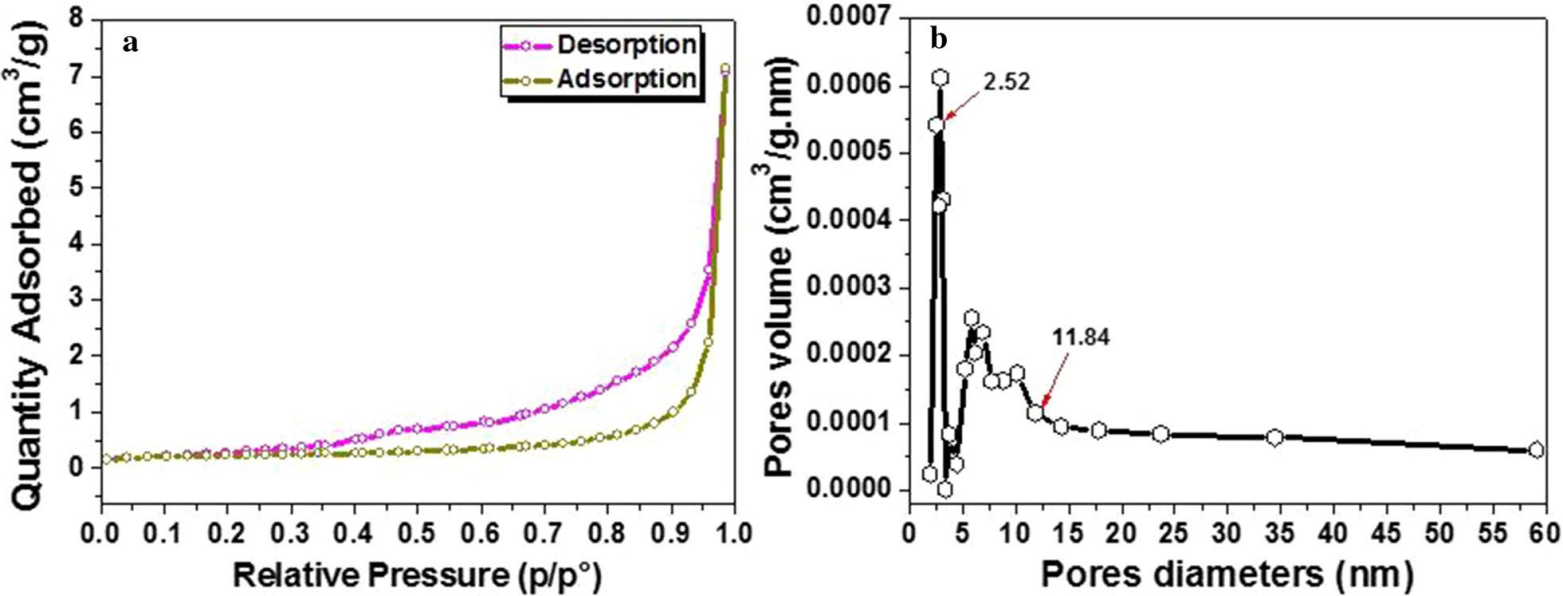
Nitrogen adsorption–desorption isotherm of the Na_2_PdP_2_O_7_ (**a**), BJH pore size distribution (**b**)

The surface area of Na_2_PdP_2_O_7_ was determined by BET method fromthe nitrogen adsorption–desorption. The BET surface area of the Na_2_PdP_2_O_7_ catalyst was found to be 1.16 m^2^/g. Indeed, the N_2_ adsorption–desorption isotherm shown in Fig. 8a exhibited isotherm type IV according to the IUPAC classification with a distinct hysteresis loop of H2. The BJH pore size distribution (Fig. 10b) revealed that the Na_2_PdP_2_O_7_ catalyst exhibits a mesoporous character with the presence of three pore size distribution peaks ranging between 2.52 and 11.84 nm.

### Catalytic activity evaluation

**Scheme 2:**
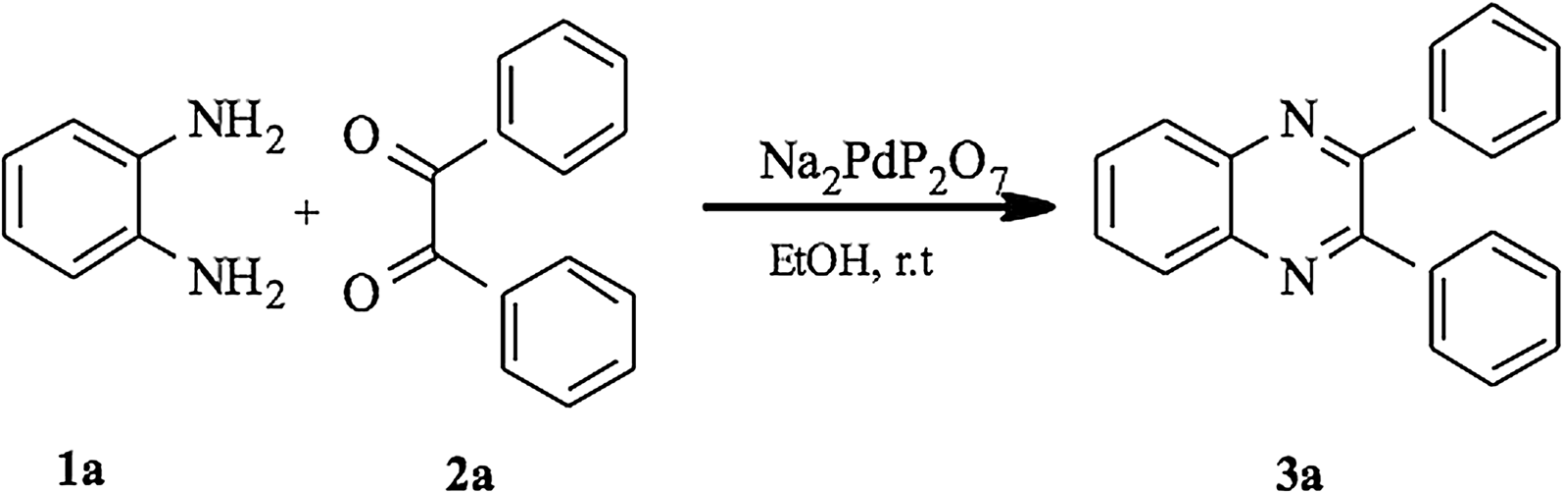
Condensation reaction of benzil and 1,2-diaminobenzene using a catalytic amount of nanostructured Na_2_PdP_2_O_7_

To investigate the catalytic activity of Na_2_PdP_2_O_7_ in the condensation reaction, we have studied the model reaction of benzene-1,2-diamine **1a** with benzyl **2a** using ethanol as the solvent in the presence of the nanostructured Na_2_PdP_2_O_7_ catalyst (Scheme 2).

**Fig. 9:**
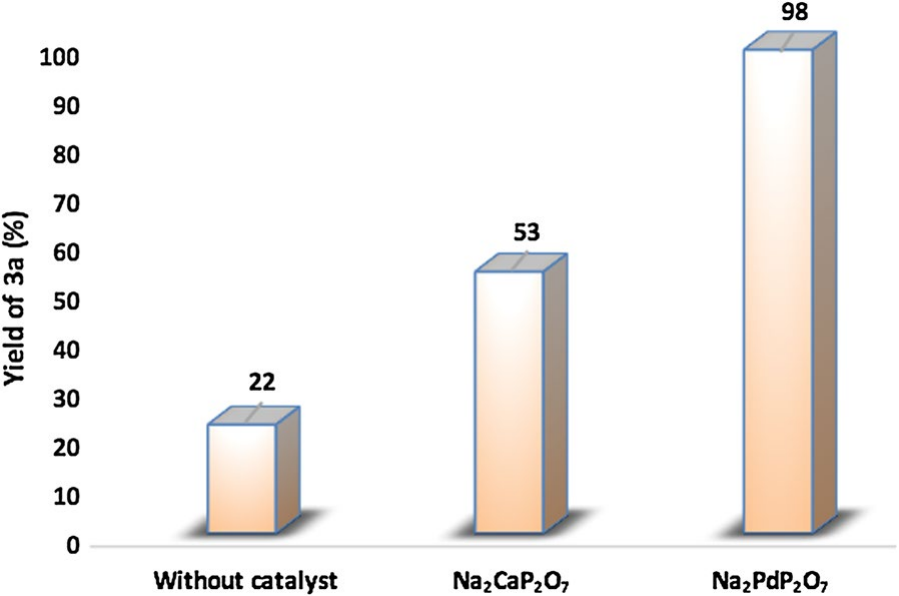
Evaluation and screening of catalysts on condensation of o-phenylenediamine with benzil

The preliminary experiments were started by screening the activities of some samples. The obtained results of these exploratory experiments are summarized in Fig. 9. Since the

2-diaminobenzene **1a** and benzyl **2a** are very reactive, the condensation reaction between **1a** and **2a** was also carried out without a catalyst under the following reaction condition: 3 mL of ethanol as solvent and at room temperature. As shown in Fig. 9, when the reaction was conducted without a catalyst, the reaction rate was very slow and the yield of **3a** did not exceed 22%. Moreover, the Na_2_CaP_2_O_7_ catalyst showed a low catalytic activity, giving only 53% conversion after 30 min. However, using Na_2_PdP_2_O_7_ as catalyst gives nearly complete conversion and yielded 98% within 30 min. This result shows the importance of this catalytic system developed in this work.

**Fig. 10:**
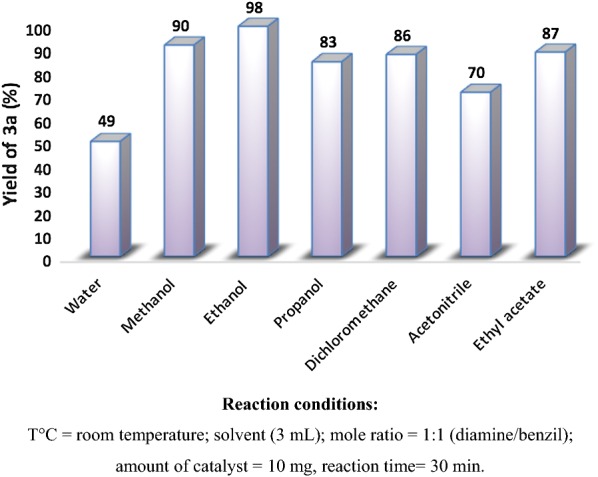
Effect of different solvents on the quinoxaline yield

The effect of various parameters, namely: Temps, nature and volume of the solvent were investigated. Initially we investigated there action of 1,2-diaminobenzene and benzyl over the Na_2_PdP_2_O_7_ catalyst in the presence of various solvents namely water, dichloromethane, acetonitrile, ethyl acetate, methanol, propanol and ethanol. The effect of various protic and aprotic solvents on the yield of quinoxaline is depicted in Fig. 10. As shown in this figure, the reaction proceeded comparatively well in aprotic solvent such as dichloromethane (86%), ethyl acetate (87%) and acetonitrile (70%). Among the solvents examined, protic solvents such as alcohols were found to be suitable solvents for quinoxaline synthesis. Excellent yields of the product **3a** were obtained when using 3 mL of propanol (83%) methanol (90%) and ethanol (98%). In the case of water, we obtained moderate yields 49%. This can be explained by the low solubility of the organic substrates in water. According to these results, ethanol was considered as the best solvent because of it effective and greener in nature for further studies.

**Fig. 11:**
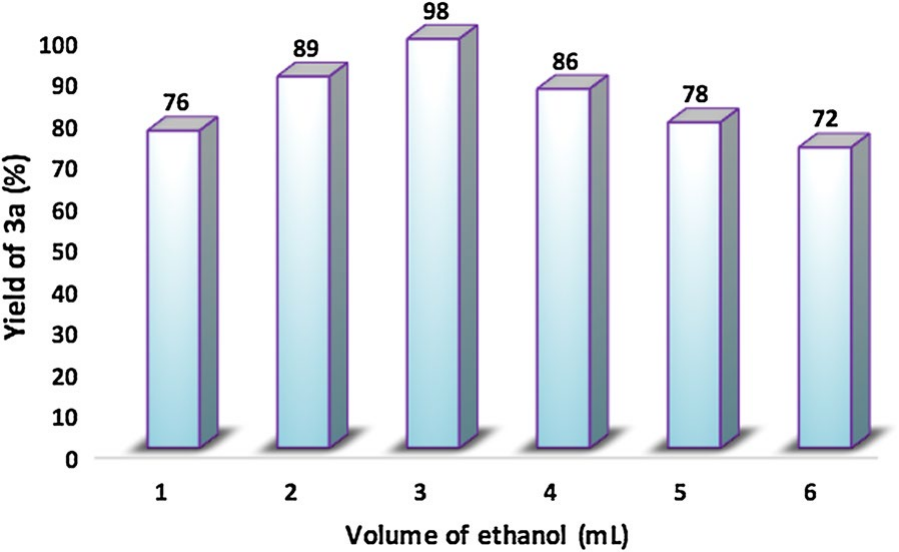
Effect of the volume of ethanol in the synthesis of quinoxaline **3a**

To investigate the effect of the solvent volume on the yield of quinoxaline, the reaction of 1,2-diaminobenzene with benzil was performed at room temperature using 10 mg of catalyst and different volume of ethanol over a period of 30 min, the results are presented in Fig. 11. As can be seen from this Figure, the quinoxaline yield increased drastically with increasing volume of ethanol until an optimum value of 3 mL and then decreased gradually. Indeed, when the volume of ethanol was increased from 1 to 3 mL, the reaction yield increased from 76 to 98%. However, further increase in the volume of the ethanol up to 6 mL resulted in a significant drop in the quinoxaline yield (72% yield for 6 mL). This drop-in product yield can probably be due to the dilution phenomenon and to the high dispersion of the reagents when large volume of ethanol was used.

**Fig. 12:**
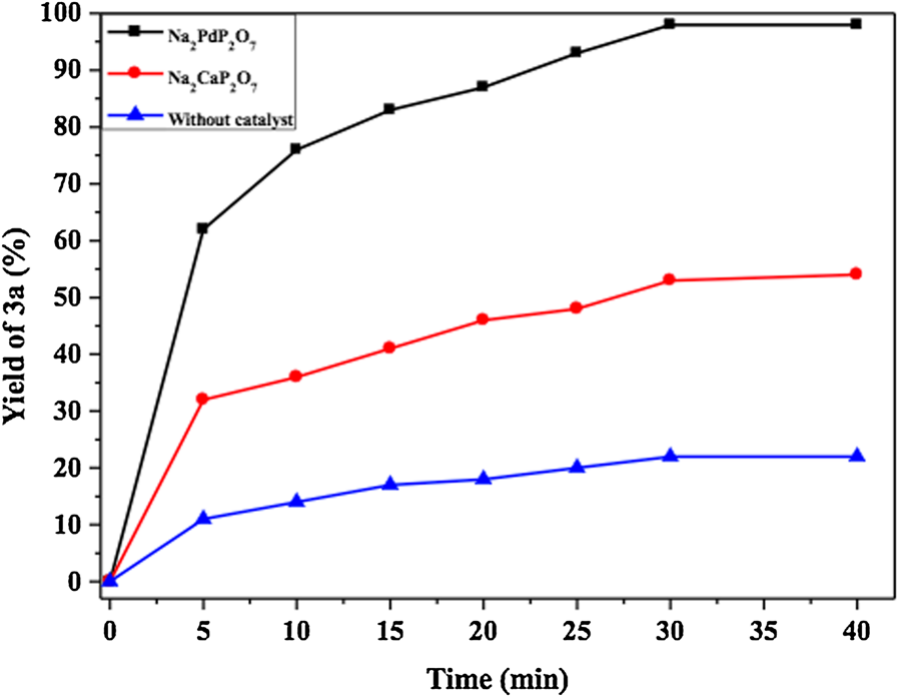
Kinetic study of the synthesis of quinoxaline **3a** catalyzed by nanostructured Na_2_PdP_2_O_7_, Na_2_CaP_2_O_7_and without catalyst, respectively

The effect of the reaction time was also investigated from 5 to 40 min (Fig. 12). As shown in this figure, the nanostructured Na_2_PdP_2_O_7_ is the best catalyst compared with Na_2_CaP_2_O_7_ and in the absence of any catalyst. When the reaction time was 5 min, the yield was modest (62%). The increase in reaction time induced a significant increase in yield. The optimal time for the condensation of *o*-phenylenediamine with benzyl is 30 min, over this period of time, the yield does not evolve anymore.

Encouraged by the remarkable results obtained with the above optimized reaction conditions, we looked at examine the utility of this methodology in order to generalize the reaction with various substituted 1,2-diamine and 1,2-dicarbonyl compounds over the prepared Na_2_PdP_2_O_7_ catalyst; the obtained results are summarized in Table 1. As illustrated in this Table, most of the reactions preceded very effectively at room temperature and no undesirable side-reactions were observed, although the yields were highly dependent on the substrate used. Indeed, the presence of electron-donating substituent such as methyl group on benzene-1,2-diamine substrate did not affect the reaction time and thus no significant difference in quinoxaline derivative yield was observed (Entry 2), while electron withdrawing substituents present in the benzene-1,2-diamines substrate (Entries 3 and 4) decreased the rate of reaction notably, and the corresponding yields were also low as compared to unsubstituted benzene-1,2-diamine. On the other hand, the reaction between aliphatic 1,2-dicarbonyl compounds such as biacetyl (Entries 5–8) with benzene-1,2-diamine containing electron-donating groups such as methyl group provided a good yield (Entry 6), while electron withdrawing substituent gave a satisfactory yield (Entry 7–8). According to the obtained results, we noticed that the aliphatic carbonyls substrates are less reactive than aromatic diketones.

**Table 1:** Synthesis of quinoxaline derivatives using nanostructured Na_2_PdP_2_O_7_

Entry	Amine	1,2-Dicarbonyl	Product	Yield^a^ (%)
1				98
2				95
3				81
4				86
5				91
6				86
7				77
8				80

Reaction condition: diketone (1 mmol), diamine (1 mmol), 10 mg of Na_2_PdP_2_O_7_, ethanol (3 mL) and stir rt

^a^Isolated yield

**Scheme 3:**
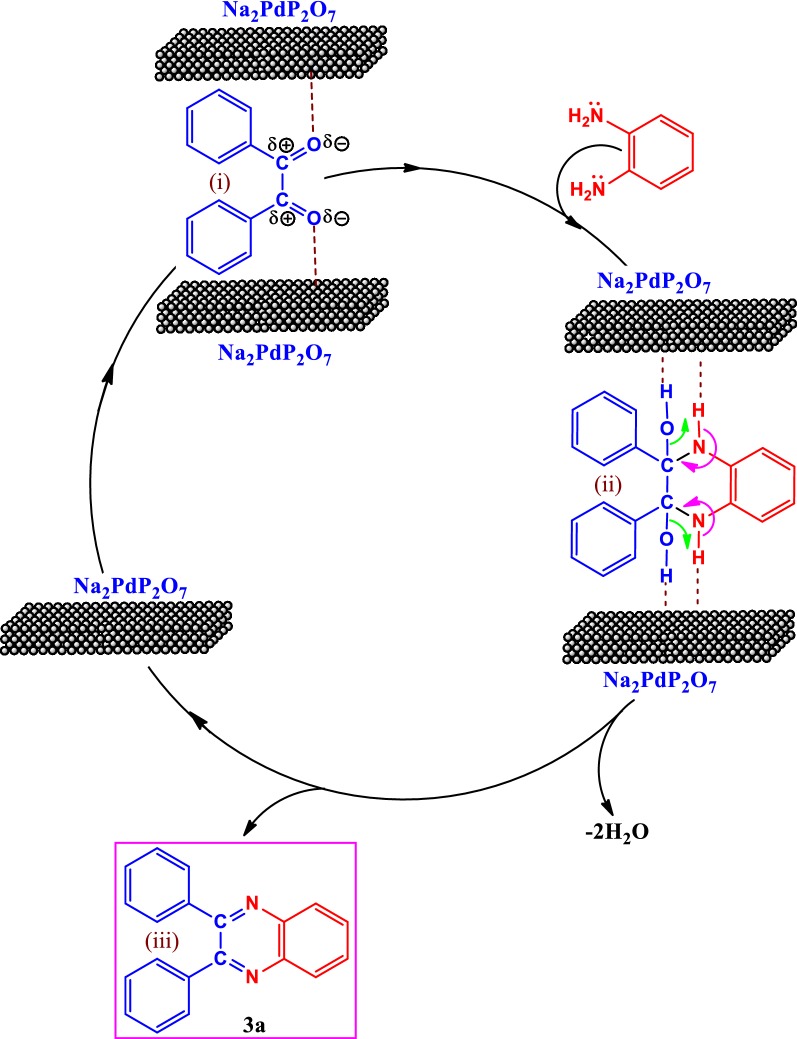
A proposed mechanism for the synthesis of 2,3-diphenylquinoxaline **3a**

One of the key points to understand the reaction mechanism in heterogeneous catalysis is the determination of the active catalytic sites. The Na_2_PdP_2_O_7_is expected to be a bifunctional catalyst owing to the presence of both acid and basic sites such as P_2_O_7_^4−^, PO_4_^3−^, Na^+^and Pd^2+^. We suggest that the condensation reaction occurs over both acid sites and basic sites involved in the Na_2_PdP_2_O_7_ catalyst. The plausible mechanism for this reaction was proposed in Scheme 3. The reaction mechanism occurs in three steps: 1,2-diketone was initially activated by the acidic sites of the Na_2_PdP_2_O_7_ catalyst (i). Afterward, nucleophilic attack by the amino group involved in the benzene-1,2-diamine on the activated 1,2-diketones generated the 2,3-diphenyl-1,2,3,4-tetrahydro-quinoxaline-2,3-diol as an intermediate (ii); internal rearrangement, followed by elimination of two water molecules, resulted in the formation of the quinoxaline **3a** (iii).

**Fig. 13:**
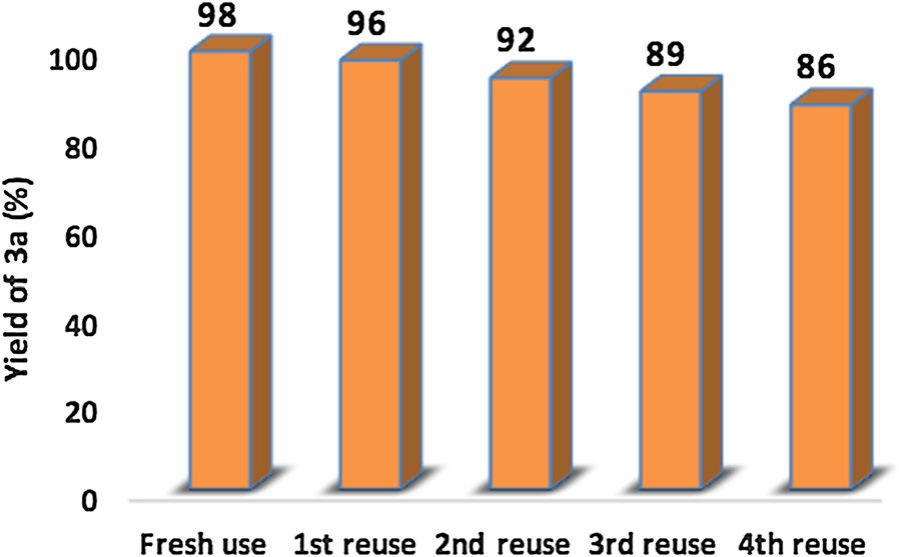
Reuse performance of the nanostructured Na_2_PdP_2_O_7_in the synthesis of quinoxaline

The reusability of the catalyst is one of the most important features of a heterogeneous catalyst especially from an economic and environmental point of view. For this purpose, a recycling study of the Na_2_PdP_2_O_7_ catalyst was conducted using the condensation reaction between the benzene-1,2-diamine with benzil as a model reaction. After each cycle, the Na_2_PdP_2_O_7_ catalyst was recovered by simple filtration, washed with dichloromethane, dried at 100 °C overnight and then directly reused in the next run under similar reaction condensations. Fifth consecutive runs were performed and the obtained results are shown in Fig. 13. As can be seen, there used catalyst showed a slight decrease in its catalytic activity during the first three runs. However, a significant decrease in the quinoxaline yield was observed after five consecutive runs. This a partial deactivation of the catalyst can be explained by the adsorbed reactants on the surface of the Na_2_PdP_2_O_7_ catalyst, which poison the catalytic surface of the catalyst and hinder the reagents to access to the active sites.

## Conclusions

In conclusion, the present work propose a simple and green synthetic methodology for the synthesis of quinoxaline and its derivatives by the direct condensation of 1,2-dicarbonyl with substituted aryl 1,2-diamines, using nanostructured Na_2_PdP_2_O_7_ as a highly efficient heterogeneous catalyst. Under optimized conditions, our developed nanostructured catalyst showed high catalytic activity using ethanol as a green solvent at room temperature. The easy work-up, short reaction time, good yield of the desired products and eco-friendly process are the noteworthy features of our synthesis procedure. Furthermore, the catalyst can be easily separated from the reaction mixture and directly reused in several cycles with only a slight drop in its catalytic activity. For practical application, the heterogeneous Na_2_PdP_2_O_7_ catalyst appeared to be promising candidate to replace the conventional homogeneous and expensive heterogeneous catalysts, currently used in the synthesis of various industrially important and biologically active quinoxalines.

## Supplementary information

**Additional file 1.** Analytical and physicochemical data of quinoxaline derivatives **3a–3h**.

## Data Availability

All data generated or analysed during this study are included in this published article [and its Additional file 1].

## Abbreviations

FTIR: Fourrier Transform infrared
XRD: X-ray diffraction
TGA: Thermogravimetric analyses
SEM: Scanning electron microscope
TEM: Transmission electron microscope
IUPAC: International Union of Pure and Applied Chemistry
EDS: Energy dispersive spectroscopy
^1^HNMR: Proton nuclear magnetic resonance spectroscopy
^13^CNMR: Carbon nuclear magnetic resonance spectroscopy
CDCl_3_: Deuterochloroform
TMS: Tetramethylsilane
MAS-NMR: Magic angle spinning in solid-state NMR spectroscopy
KBr: Potassium bromide

## References

[1] Corona P, Carta A, Loriga M, Vitale G, Paglietti G (2009). Synthesis and in vitro antitumor activity of new quinoxaline derivatives. Eur J Med Chem.

[2] Kotharkar SA, Shinde DB (2006). Synthesis of antimicrobial 2,9,10-trisubstituted-6-oxo-7,12-dihydro-chromeno[3,4-b]quinoxalines. Bioorg Med Chem Lett.

[3] Ganapathy S, Ramalingam P, Baburao C (2008). Antimicrobial and antimycobacterial activity of some quinoxalines ‘N’ bridgehead heterocycles. Asian J Chem.

[4] Khan SA, Saleem K, Khan Z (2007). Synthesis, characterization and in vitro antibacterial activity of new steroidal thiazoloquinoxalines. Eur J Med Chem.

[5] Guillon J, Forfar IM, Matsuda M, Desplat V, Saliège M, Thiolat D, Massip S, Tabourier A, Léger JM, Dufaure B, Haumont G, Jarry C, Mossalayi D (2007). Synthesis, analytical behaviour and biological evaluation of new 4-substituted pyrrolo[1,2-a]quinoxalines as antileishmanial agents. Bioorg Med Chem..

[6] Kakanejadifard A, Zabardasti A, Ghasemian M, Toosi-Jamali H (2008). Synthesis and characterization of Cd(II), Zn(II) and Hg(II) chloride adducts of (2Z,3Z)-1,4,7- trithiononane-2,3-dionedioxime. Asian J Chem.

[7] Brock ED, Lewis DM, Yousaf TI, Harper HH. The Procter and Gamble Company, USA WO 9951688, 1999

[8] Thomas KRJ, Velusamy M, Lin JT, Chuen CH, Tao YT (2005). Chromophore-labeled quinoxaline derivatives as efficient electroluminescent materials. Chem Mater.

[9] Balta DK, Keskin S, Karasu F, Arsu N (2007). Quinoxaline derivatives as photoinitiators in UV-cured coatings. Prog Org Coat.

[10] Dailey S, Feast WJ, Peace RJ, Sage IC, Till S, Wood EL (2001). Synthesis and device characterisation of side-chain polymer electron transport materials for organic semiconductor applications. J Mater Chem.

[11] Antoniotti S, Duñach E (2002). Direct and catalytic synthesis of quinoxaline derivatives from epoxides and ene-1,2-diamines. Tetrahedron Lett.

[12] Shi DQ, Dou GL, Ni SN, Shi JW, Li XY (2008). An efficient synthesis of quinoxaline derivatives mediated by stannous chloride. J Heterocycl Chem.

[13] Robinson RS, Taylor RJK (2005). Quinoxaline synthesis from α-hydroxy ketones via a tandem oxidation process using catalysed aerobic oxidation. Synlett.

[14] Yadav JS, Reddy BVS, Rao YG, Narsaiah AV (2008). First example of Cu(OTf)_2_-catalyzed synthesis of quinoxalines from α-diazoketones and aryl 1,2-diamines. Chem Lett.

[15] Wu HW, Yang GS (2008). One-pot synthesis of quinoxalines from alpha-haloketones and aromatic 1, 2-diamines via an oxidation-condensation process. Chin J Org Chem..

[16] Reich BJE, Justice AK, Beckstead BT, Reibenspies JH, Miller SA (2004). Cyanide-catalyzed cyclizations via aldimine coupling. J Org Chem.

[17] Haldar P, Dutta B, Guin J, Ray JK (2007). Uncatalyzed condensation between aryl-1,2-diamines and diethyl bromomalonate: a one-pot access to substituted ethyl 3-hydroxyquinoxaline-2-carboxylates. Tetrahedron Lett.

[18] Huang TK, Wang R, Shi L, Lu XX (2008). Montmorillonite K-10: an efficient and reusable catalyst for the synthesis of quinoxaline derivatives in water. Catal Commun.

[19] Srinivas C, Kumar CNSSP, Rao VJ, Palaniappan S (2007). Efficient, convenient and reusable polyaniline-sulfate salt catalyst for the synthesis of quinoxaline derivatives. J Mol Catal Chem.

[20] Hasaninejad A, Zare A, Mohammadizadeh MR, Shekouhy M (2008). Oxalic acid as an efficient, cheap, and reusable catalyst for the preparation of quinoxalines via condensation of 1,2-diamines with α-diketones at room temperature. Arkivoc.

[21] More SV, Sastry MNV, Yao CF (2006). Cerium (IV) ammonium nitrate (CAN) as a catalyst in tap water: a simple, proficient and green approach for the synthesis of quinoxalines. Green Chem.

[22] Li Z, Li W, Sun Y, Huang H, Ouyang P (2008). Room temperature facile synthesis of quinoxalines catalyzed by amidosulfonic acid. J Heterocycl Chem.

[23] Heravi MM, Bakhtiari K, Bamoharram FF, Tehrani MH (2007). Wells-Dawson type heteropolyacid catalyzed synthesis of quinoxaline derivatives at room temperature. Monatsh Chem.

[24] Yadav JS, Reddy BVS, Premalatha K, Shankar KS (2008). Bismuth(III)-catalyzed rapid synthesis of 2,3-disubstituted quinoxalines in water. Synthesis.

[25] Hazarika P, Gogoi P, Konwar D (2007). Efficient and green method for the synthesis of 1,5-benzodiazepine and quinoxaline derivatives in water. Synth Commun.

[26] Potewar TM, Ingale SA, Srinivasan KV (2008). Efficient synthesis of quinoxalines in the ionic liquid 1-*n*-butylimidazolium tetrafluoroborate ([Hbim]BF_4_) at ambient temperature. Synth Commun.

[27] Hasaninejad A, Zare A, Zolfigol MA, Shekouhy M (2009). Zirconium Tetrakis (dodecyl Sulfate) [Zr(DS)4] as an efficient lewis acid-surfactant combined catalyst for the synthesis of quinoxaline derivatives in aqueous media. Synth Commun.

[28] Cai JJ, Zou JP, Pan XQ, Zhang W (2008). Gallium(III) triflate-catalyzed synthesis of quinoxaline derivatives. Tetrahedron Lett.

[29] Bhosale RS, Sarda SR, Ardhapure SS, Jadhav WN, Bhusare SR, Pawar RP (2005). An efficient protocol for the synthesis of quinoxaline derivatives at room temperature using molecular iodine as the catalyst. Tetrahedron Lett.

[30] Guo WX, Jin HL, Chen JX, Chen F, Ding JC, Wu HY (2009). An efficient catalyst-free protocol for the synthesis of quinoxaline derivatives under ultrasound irradiation. J Braz Chem Soc.

[31] Zhao Z, Wisnoski DD, Wolkenberg SE, Leister WH, Wang Y, Lindsley CW (2004). General microwave-assisted protocols for the expedient synthesis of quinoxalines and heterocyclic pyrazines. Tetrahedron Lett.

[32] Oveisi H, Adharvana MC, Chi VN, Jeffrey EC, Saad MA, Ekrem Y, Shahriar AHM, Yusuke Y, Kevin CWW (2017). ZnO-loaded mesoporous silica (KIT-6) as an efficient solid catalyst for production of various substituted quinoxalines. Catal Commun.

[33] Ajeet K, Santosh K, Amit S, Arnab D, Subho M (2008). Ni-nanoparticles: an efficient catalyst for the synthesis of quinoxalines. Catal Commun.

[34] Fan LY, Wei L, Hua WJ, Li XX (2014). Yb modified NaY zeolite: a recyclable and efficient catalyst for quinoxaline synthesis. Chin Chem Lett.

[35] Jafarpour M, Rezaeifard A, Danehchin M (2011). Easy access to quinoxaline derivatives using alumina as an effective and reusable catalyst under solvent-free conditions. Appl Catal A Gen.

[36] Roy B, Ghosh S, Ghosh P, Basu (2015). Graphene oxide (GO) or reduced graphene oxide (rGO): efficient catalysts for one-pot metal-free synthesis of quinoxalines from 2-nitroaniline. Tetrahedron Lett.

[37] Sadjadi S, Sadjadi S, Hekmatshoar R (2010). Ultrasound-promoted greener synthesis of benzoheterocycle derivatives catalyzed by nanocrystalline copper(II) oxide. Ultrason Sonochem.

[38] Mirjalili BBF, Akbari A (2011). Nano-TiO_2_: an eco-friendly alternative for the synthesis of quinoxalines. Chin Chem Lett.

[39] Sato Y, Shen Y, Nishida M, Kanematsu W, Hibino T (2012). Proton conduction in non-doped and acceptor-doped Metal pyrophosphate (MP_2_O_7_) composite ceramics at intermediate temperatures. J Mater Chem.

[40] Zhang YC, Cheng W, Wu D, Zhang H, Chen D, Gong Y, Kan Z (2004). Crystal and band structures, bonding, and optical properties of solid compounds of alkaline indium (III) pyrophosphates MInP2O7(M = Na, K, Rb, Cs). Chem Mater.

[41] Inoue S, Nobuyuki O. Stationary phase material for chromatography. US 5728463 A, 1988

[42] Dânoun K, Jioui I, Bouhrara M, Zahouily M, Solhy A, Jouiad M, Len C, Fihri A (2015). Nano-structured pyrophosphate Na_2_CaP_2_O_7_ as catalyst for selective synthesis of 1,2-disubstituted benzimidazoles in pure water. Curr Org Chem.

[43] Jioui I, Dânoun K, Solhy A, Jouiad M, Zahouily M, Essaid B, Len C, Fihri A (2016). Modified fluorapatite as highly efficient catalyst for the synthesis of chalcones via Claisen-Schmidt condensation reaction. J Ind Eng Chem.

[44] Dânoun K, Essamlali Y, Amadine O, Tabit R, Fihri A, Len C, Zahouily M (2018). Nanostructured pyrophosphate Na2PdP2O7-catalyzed Suzuki-Miyaura cross-coupling under microwave irradiation. Appl Organomet Chem.

[45] Perry DL, Phillips SL (1998). Handbook of inorganic compounds.

