# Supplementary material for: Eco-friendly approach to access of quinoxaline derivatives using nanostructured pyrophosphate Na2PdP2O7 as a new, efficient and reusable heterogeneous catalyst
Source: BMC Chem. 2020 Feb 3;14(1):6. doi: 10.1186/s13065-020-0662-z (PMC6996184; doi:10.1186/s13065-020-0662-z)
Supplement: Supplementary file 1 — Additional file 1. Analytical and physicochemical data of quinoxaline derivatives 3a–3h. [file 13065_2020_662_MOESM1_ESM.docx]

**Eco-friendly Approach to Access of Quinoxaline Derivatives Using Nanostructured Pyrophosphate Na_2_PdP_2_O_7_ as a New, efficient and Reusable Heterogeneous Catalyst**

Karim Dânoun,**^1,2^** Younes Essamlali,**^1^** Othmane Amadine,**^1^** Hassan Mahi,**^1^** Mohamed Zahouily**^1,2*^**

**^1^**Moroccan Foundation for Advanced Science, Innovation and Research (MAScIR), VARENA Center, Rue Mohamed El Jazouli, Madinat Al Irfane, 10100 Rabat,Morocco.

**^2^**University Hassan II Casablanca, FST Mohammedia, Laboratory of Materials, Catalysis and Valorization of Natural Resources - URAC 24, B.P. 146, 20650, Morocco.

***Correspondence:** m.zahouily@mascir.com

***Correspondence:** m.zahouily@mascir.com

**ADDITIONAL FILE 1**

**Analytical and physicochemical data of quinoxaline derivatives 3a-3h.**

- **2,3-Dimethylquinoxaline (3a).**

Colour: White solid; Mp126-128°C. TLC (10% EtOAc/Hex) Rf 0.45. ^1^H NMR (600 MHz, CDCl_3_):δ (ppm) 8.18 (dd. 2 H. J=3.28 ; 9.83 Hz); 7.91 (dd. 2 H.J = 3.28 ; 9.83 Hz); 7.49 (d. 4 H. J = 7.10 Hz); 7.41 (m. 6 H).^13^C NMR (151 MHz. CDCl_3_): δ (ppm) 153.51; 141.25; 139.07; 130.03; 129.86; 192.68;129.23; 128.86; 128.71; 128.43; 128.33.IR (KBr) 3065 ;1441cm^-1^. HRMS calculated for C_20_H_14_N_2_ (M+H)^+^ 283.1230; found: 283.1234.

- **6-Methyl-2,3-diphenylquinoxaline (3b).**

Colour: White solid. Mp 112-114°C.TLC (10% EtOAc/Hex) Rf 0.48. ^1^H NMR (600 MHz, CDCl_3_):δ (ppm)8.09 (s. 1 H); 8.08 (s. 1 H); 7.97 (m. 1 H); 7.63 (m. 1 H); 7.53 (dd. 4 H. J=5.01; 7.48 Hz); 7.39 (m. 6 H); 2.64 (s. 3 H).^13^C NMR (151 MHz. CDCl_3_): δ (ppm)153.36; 152.60; 141.30; 140.55; 139.72; 139.21; 132.37; 129.95; 129.86; 129.84; 129.06; 128.73; 128.71; 128.67; 128.27; 128.03; 21.98.IR (KBr)3054; 2975; 1619; 1484cm^-1^. HRMS calculated for C_21_H_16_N_2_ (M+H)^+^ 297.1386; found: 297.1391.

- **6-Chloro-2,3-diphenylquinoxaline (3c).**

Colour: White solid. Mp124-126°C. TLC (10% EtOAc/Hex) Rf 0.37. ^1^H NMR (600 MHz, CDCl_3_):δ (ppm)8.19 (s. 1 H); 8.14 (d. 1 H. J = 8.68 Hz); 7.74 (m.1H); 7.53 (d. 4 H. J= 6.98 Hz); 7.40 (m. 6 H).^13^C NMR (151 MHz. CDCl_3_): δ (ppm)154.51; 153.68; 141.42; 139.65; 138.91; 135.69;131.02; 130.45; 129.95; 129.85; 129.80; 129.15; 129.06; 128.09.IR (KBr)3055; 1606; 1497cm^-1^. HRMS calculated for C_20_H_13_ClN_2_ (M+H)^+^ 317.0840; found: 317.0851.

- **6-Bromo-2,3-dimethylquinoxaline (3d).**

Colour: White solid. Mp120-122°C.TLC (10% EtOAc/Hex) Rf 0.28. ^1^H NMR (600 MHz, CDCl_3_):δ (ppm)8.38 (s. 1 H); 8.07 (m. 1 H); 7.87 (d. 1 H. J =
7.29 Hz); 7.55 (m. 4 H); 7.40 (m. 6 H).^13^C NMR (151 MHz. CDCl_3_): δ (ppm)154.02; 153.78; 141.78; 139.96; 138.71; 138.60; 130.53; 129.95; 129.85; 129.16; 129.06; 128.38; 128.36; 123.88.IR (KBr)3055; 1593; 1445cm^-1^. HRMS calculated for C_20_H_13_BrN_2_. (M+H)^+^ 361.0840; found: 361.0851.

- **2,3-dimethylquinoxaline (3e).**

Colour: White solid. Mp102-104°C.TLC (10% EtOAc/Hex) Rf0.55. ^1^H NMR (600 MHz. CDCl_3_): δ (ppm) 8.01 (dd. 2 H. J = 3; 9.42 Hz); 7.70 (m. 2 H); 2.76 (s. 6 H).^13^C NMR (151 MHz. CDCl_3_): δ (ppm) 153.82; 153.54; 141.05; 140.92; 128.92; 128.29;23.22.IR (KBr)2998; 1564; 1484; 1393cm^-1^.HRMS calculated for C_10_H_10_N_2_. (M+H)^+^159.0845; found: 159.0855.

- **2,3,6-trimethylquinoxaline (3f).**

Colour: Yellow solid. Mp91-93°C.TLC (10% EtOAc/Hex) Rf0,52. ^1^H NMR (600 MHz. CDCl_3_): δ (ppm)7.88 (d. 1 H. J = 8.48 Hz); 7.76 (s. 1 H); 7.52 (d.
1H. J = 8.48 Hz); 2.72 (s. 6 H); 2.57 (s. 3 H).^13^C NMR (151 MHz. CDCl_3_): δ (ppm) 153.37; 152.50; 141.07; 139.42; 139.26; 131.13;127.76; 127.24; 23.19; 23.08; 21.79.IR (KBr)3008; 1616; 1441; 1322cm^-1^. HRMS calculated for C_11_H_12_N_2_. (M+H)^+^173.2335; found: 173.2343.

- **6-chloro-2,3-dimethylquinoxaline (3g).**

Colour: Yellow solid. Mp89-91°C.TLC (10% EtOAc/Hex) Rf0.42. ^1^H NMR (600 MHz. CDCl_3_): δ (ppm)7.98 (s. 1 H); 7.93 (d. 1 H. J = 8.66 Hz); 7.63 (m.1 H); 2.74 (s. 6 H).^13^C NMR (151 MHz. CDCl_3_): δ (ppm) 154.66; 153.85; 141.31; 139.49; 134.81; 129.89;128.94; 127.30; 25.66; 23.20.IR (KBr)1604; 1481; 1325cm^-1^.HRMS calculated for C_10_H_9_ClN_2_. (M+H)^+^193.0420; found: 193.0436.

- **6-bromo-2,3-dimethylquinoxaline (3h).**

Colour: Yellow solid. Mp 93-95°C. TLC (10% EtOAc/Hex) Rf 0.35.^1^H NMR (600 MHz. CDCl_3_): δ (ppm)8.17 (s. 1 H); 7.87 (d. 1 H. J = 8.76 Hz.); 7.76 (m. 1 H); 2.74 (s. 6 H).^13^C NMR (151 MHz. CDCl_3_): δ (ppm) 154.29; 153.60; 141.31; 139.43; 134.82; 129.89;128.94; 127.30; 25.27; 23.60.IR (KBr)3085; 2950; 1597; 1480cm^-1^.HRMS calculated for C_10_H_9_ClN_2_. (M+H)^+^236.9942; found: 236.9955.
